# Hemoglobin-to-Red Cell Distribution Width Ratio as a Predictor of Gastrointestinal Bleeding Following Percutaneous Coronary Intervention

**DOI:** 10.1155/crp/2793810

**Published:** 2025-04-27

**Authors:** Ting Zhang, Yun Wang, Xuemei Su, Yangqing Liu

**Affiliations:** Longyan First Affiliated Hospital of Fujian Medical University, Longyan 364000, China

**Keywords:** coronary heart disease, gastrointestinal bleeding, hemoglobin-to-red-cell distribution width ratio, percutaneous coronary intervention

## Abstract

**Background:** Many patients with coronary heart disease receive percutaneous coronary interventions. These interventions are accompanied by gastrointestinal bleeding that aggravates the disease. The hemoglobin-to-red cell distribution width ratio (HRR) is a novel inflammatory marker. We investigated HRR as a predictor of gastrointestinal bleeding after percutaneous coronary interventions.

**Methods:** Patients (*n* = 1647) received percutaneous coronary interventions from January 2022 to December 2022 in Longyan First Hospital. The HRR was measured before the interventions. Indicators of patient general condition, biochemical indicators, concomitant diseases, and medication status were collected. Gastrointestinal bleeding within 1 year was assessed. Patients were divided into four groups based on HRR. Kendall's tau-b graded correlation was used to analyze the correlation between hemoglobin (Hb), red blood cell distribution width (RDW), HRR, and gastrointestinal bleeding in peripheral blood after percutaneous coronary intervention. Ordered logistic regression was used for analysis, with gastrointestinal bleeding as the outcome variable and Hb, RDW, and HRR as independent variables. To identify independent risk factors for gastrointestinal bleeding, data were adjusted for age, heart failure, hypertension, diabetes, atrial fibrillation, dyslipidemia, RBC, total cholesterol, triglycerides, LDL-C, creatinine, blood urea nitrogen, and uric acid. Multiple linear regression analysis of HRR, RDW, and Hb predicted gastrointestinal bleeding.

**Results:** Of the 1647 study participants, 20 had gastrointestinal bleeding, 1.2% probability. In the HRR classification, there was a greater probability of gastrointestinal bleeding in the low HRR group after percutaneous coronary intervention.

**Conclusion:** We found a low HRR and a high probability of gastrointestinal bleeding after percutaneous coronary intervention. The HRR could be used as an independent predictor of gastrointestinal bleeding.

## 1. Introduction

Coronary heart disease (CHD) is a common and often fatal heart disease. The incidence of CHD is increasing yearly [1–3]. CHD has become the world's largest cause of death [4, 5]. Some patients who undergo percutaneous coronary intervention (PCI) are at risk of postoperative complications. Gastrointestinal bleeding is a frequent adverse event in the first year after PCI and usually requires temporary interruption of antiplatelet therapy to identify and treat the sources of bleeding [6]. The probability of gastrointestinal bleeding after PCI is about 1% [7–10]. Gastrointestinal bleeding is a common disease that may lead to substantial morbidity and mortality [11–13].

Antiplatelet therapy after PCI combined with gastrointestinal bleeding is a condition that not only aggravates CHD but also the condition may lead to hospitalization, increased cost of treatment, and increased mortality compared with the absence of gastrointestinal bleeding [4, 7–10, 14]. Thus, there is a great need for methods to predict and prevent the occurrence of gastrointestinal bleeding after PCI.

Hemoglobin-to-red-cell distribution width ratio (HRR) is closely associated with inflammation, age, decline, malnutrition, and renal insufficiency [15–18]. These associations make HRR an indicator [7, 19] that can predict an elevated incidence and increased mortality of CHD. Thus, we reasoned that HRR would be an effective predictor of gastrointestinal bleeding after PCI.

HRR is an important index in routine blood testing, with a simple measurement method and low cost, suitable for all patients. Therefore, we expect that HRR would be an effective indicator for predicting gastrointestinal bleeding after PCI, hence helping physicians to recognize possible postoperative complications in advance.

## 2. Materials and Methods

### 2.1. Ethical Review

The Ethics Committee of the Longyan First Affiliated Hospital of Fujian Medical University approved this study (approval number LYRE2022-K030-01). All data were obtained from the Hospital database; thus, signed informed consent was waived by the Ethics Committee.

The study inclusion criteria were the following: ① participant met the diagnostic criteria for CHD, which was confirmed by coronary angiography; ② met PCI indications; ③ age ≥ 18 years; and ④ completed the study.

The study exclusion criteria were the following: ① patients with hematologic disease, malignancy, upper gastrointestinal bleeding, or peptic ulcer before admission; ② gastric varices; ③ chronic kidney disease; and ④ mental disorder.

The study was single-center and prospective. Data were obtained for patients who underwent PCI from January 2022 to December 2022 in Longyan First Affiliated Hospital of Fujian Medical University. The number of candidates was 1654, including 1222 men (73.9%); seven patients were lost to follow-up, and a total of 1647 people (1218 men) completed the study.

### 2.2. Sociodemographic Characteristics

Sociodemographic characteristics included sex, age, body mass index, and comorbidities such as hypertension, diabetes, cardiac insufficiency, atrial fibrillation, dyslipidemia, and anemia (Table 1).

### 2.3. Peripheral Blood Parameters

Upon admission, patient blood parameters are entered into the Hospital's electronic information medical record system. Parameters include white blood cell count, platelets, red blood cells count, hemoglobin (Hb), red blood cell distribution width (RDW), glucose, total cholesterol, triglycerides, high-density lipoprotein cholesterol, low-density lipoprotein cholesterol, creatinine, blood urea nitrogen, and uric acid (Table 2).

Among these blood parameters, Hb and RDW are two key indicators. To better assess the health of the patient, the corrected Hb concentration (HRR) was also calculated. The calculation formula for HRR is HRR = Hb (g/L)/RDW (fL). This index more accurately reflects a patient's actual oxygen transport capacity and can help doctors to make a more comprehensive assessment of the patient's condition.

### 2.4. Delimiting

Gastrointestinal bleeding may be upper gastrointestinal bleeding, such as coffee-like vomiting or endoscopy showing active bleeding, or lower gastrointestinal bleeding, such as black stool, hematochezia, or endoscopic examination of active bleeding sites.

Myocardial infarction refers to an increase in the total amount of creatine kinase to twice or more the normal upper limit or an increase in the concentration of creatine kinase isoenzyme to 20 ng/mL, accompanied by symptoms and/or an ischemic electrocardiogram.

Acute kidney injury represents a 1.5-fold increase in blood creatinine concentration from the baseline.

### 2.5. Data Analysis

All data entry was performed using SPSS 27.0 (SPSS Inc., Chicago, Illinois, USA). Measurement data are expressed as the mean ± standard deviation or median, and counting data are expressed as the frequency, composition ratio, and rate. Measurement data were subjected to *t*-test, counting data were assessed by χ^2^ test, and AONVA was used to assess measurement data between multiple groups.

The association of Hb, RDW, and HRR with gastrointestinal bleeding was analyzed using Kendall's tau-b grade correlation after PCI. Analysis was performed using ordinal logistic regression, with gastrointestinal bleeding as the outcome variable and Hb, RDW, and HRR as independent variables. Independent risk factors for gastrointestinal bleeding were identified after adjusting for age, sex, heart failure, hypertension, diabetes, atrial fibrillation, dyslipidemia, white blood cells, white blood cells, red blood cells, platelets, Hb, total cholesterol, triglycerides, LDL-C, creatinine, BUN, and uric acid.

A logistic regression model was used to analyze the relation between HRR, RDW, and Hb and gastrointestinal bleeding within 1 year of discharge after PCI. The predictive efficacy of the risk assessment model was evaluated with a receiver operating characteristic curve. The strength of the association was expressed as the odds ratio (OR; 95% confidence interval), and *p* < 0.05 indicated statistical significance.

## 3. Results

### 3.1. Association Between Sociodemographic Characteristics and Gastrointestinal Bleeding After PCI

Univariate analysis of sociodemographic characteristics showed that age (*p* < 0.001), diastolic blood pressure (*p* < 0.001), acute myocardial infarction (*p*=0.017), heart failure (*p* < 0.001), hypertension (*p* < 0.001), diabetes (*p* < 0.001), atrial fibrillation (*p* < 0.001), dyslipidemia (*p* < 0.001), and acute kidney injury (*p* < 0.001) were related with statistical significance to gastrointestinal bleeding after PCI.

### 3.2. The Relation Between Blood Parameters and Gastrointestinal Bleeding After PCI

Univariate analysis of blood parameters showed that white blood cells (*p* < 0.001), red blood cells (*p*=0.027), platelets (*p*=0.0019), total cholesterol (*p* < 0.001), triglycerides (*p* < 0.001), LDL-C (*p* < 0.001), creatinine (*p* < 0.001), urea UN (*p* < 0.001), and uric acid (*p*=0.0082) were associated with gastrointestinal bleeding after PCI. In addition, Table 3 indicates that patients with a lower HRR (*p* < 0.001) had a higher probability of gastrointestinal bleeding.

Ordered logistic regression analysis was performed in which gastrointestinal bleeding (no GI bleeding and GI bleeding) was considered the dependent variable and Hb, RDW, and HRR were independent variables. The unadjusted Model 1 showed that low Hb (*p* < 0.001), low HRR (*p* < 0.001), and a high RDW (*p* < 0.001) were associated with gastrointestinal bleeding risk factors. In Model 2, age, DBP, AMI; CHF, AF, DM, hypertension, dyslipidemia, anemia, AKI, WBC, RBC, Platelet, TC, LDL-C, serum creatinine, BUN, uric acid, low Hb (*p*=0.031) were risk factors for gastrointestinal bleeding, and low HRR (*p*=0.004) was a risk factor for gastrointestinal bleeding.

## 4. Discussion

To our knowledge, this study is the first to measure the correlation between HRR and postoperative gastrointestinal bleeding after PCI. Our findings suggest that a low HRR is an important predictor of bleeding after PCI. There was a direct incremental relation between low HRR and clinical outcome.

Table 4 indicates that Hb, RDW, and HRR were associated with gastrointestinal bleeding.

HRR (Hb/RDW) is a clinical parameter that can be easily obtained by a standard blood cell count.

A decrease in HRR may be related to a decrease in Hb. The decrease in Hb level will lead to less oxygen transport capacity, which can affect the normal operation of various organs. A prolonged hypoxic state may lead to tissue damage, reduced organ function, and may even be life-threatening [20].

We found that HRR was not only associated with a decrease of Hb but also HRR was associated with an increase in RDW. However, the RDW increase reflects the uneven distribution of RBC sizes. This imbalance may be due to physiological conditions such as inflammation and oxidative stress [15–18].

Thus, the reduction in HRR can be seen as a combined response to these undesirable factors. In practical clinical work, healthcare professionals can assess the health status of their patients by monitoring Hb level and the width of the red blood cell distribution.

Table 5 suggests that low Hb, high RDW, and low HRR are independent risk factors for gastrointestinal bleeding after PCI.

Hemoglobin is the main component of the RBC, which is responsible for transporting oxygen to all organs and maintaining normal physiological function. RDW is the width of red blood cell distribution, reflecting the dispersion of red blood cell volume, which is closely related to the degree and type of anemia. After PCI, anemia is prevalent because of factors such as surgical stress, blood loss, and red blood cell destruction. A low Hb level means a decreased oxygen transport capacity, which may lead to tissue hypoxia, cause damage to the gastrointestinal mucosa, increase the risk of bleeding, and affect postoperative rehabilitation [20]. Gultekingil et al. [21] showed that patients who had anemia at presentation were prone to have significant gastrointestinal bleeding. In addition, in a study of 5018 patients with PCI, Faggioni et al. [22] found that anemia was associated with significantly higher gastrointestinal bleeding. Liao et al. [23] found that elevated RDW increases the risk of GIB after coronary artery bypass grafting surgery.

Table 3 shows that our study also identified anemia-related gastrointestinal bleeding in patients after PCI.

A high RDW indicates RBC volume instability, reflecting the imbalance of erythropoiesis and destruction. A high RDW is [15–18] associated with inflammation, oxidative stress, dyslipidemia, and abnormal vitamin D metabolism, thereby increasing the risk of gastrointestinal bleeding. All of the conditions can affect the postoperative quality of life and increase the risk of gastrointestinal bleeding.

**Figure 1:**
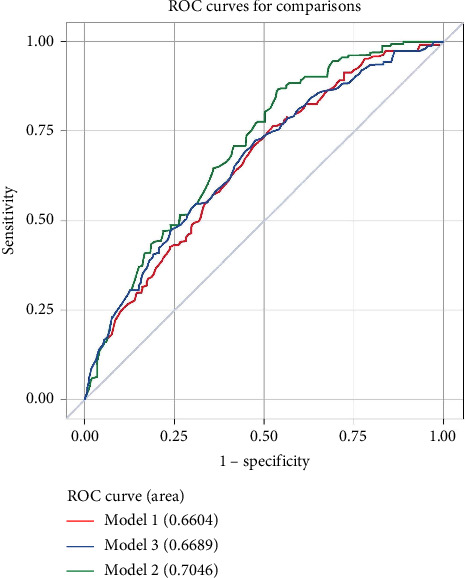
Subject operating characteristic curve for HRR and model for GI bleeding after PCI. HRR has good predictive value and was superior to Hb and RDW. HRR (Model 2): AUC = 0.7046, *p* < 0.0001; hemoglobin (Model 1): AUC = 0.6604, *p* < 0.0001; RDW (Model 3): AUC = 0.6689, *p* < 0.0001.

Figure 1 suggests that HRR is a better value for predicting gastrointestinal bleeding after PCI compared with Hb and RDW. Tapaskar found that Hb level predicted gastrointestinal bleeding [24]. However, a study by Marti showed that Hb also predicted [25] GI bleeding after PCI. In addition, RDW is a valid predictor of many pathological conditions, such as cardiovascular disease [26, 27], sepsis [28–30], and ischemic stroke [31]. HRR is closely related to inflammation, age, decline, malnutrition, and renal dysfunction [15–18]. All of these conditions may lead to a higher probability of gastrointestinal bleeding. Our predictive model suggested that HRR was a better value than Hb and RDW in predicting gastrointestinal bleeding after PCI. HRR may be a combined response to these adverse factors, thereby being superior to the separate factors Hb and RDW.

## 5. Limitation

Our study had limitations. First, the study was performed in a single center with a small number of patients; thus, there may have been selection bias. Second, the short follow-up time does not help to explain the dynamic changes of HRR. Third, HRR affects many factors, such as infection and arrhythmia, which also have significant associations. Fourth, the RDW levels may be influenced by neurohumoral activation, thyroid disease, nutritional deficiencies of folate, vitamin B12, and iron, bone marrow dysfunction, and some medications. Fifth, the RDW test values are influenced by different laboratories and reagents. In addition, the intestinal microbial community, patient anxiety, depression, and other psychological states also have an impact on gastrointestinal bleeding after PCI.

## 6. Conclusion

Our finding of lower HRR and higher probability of postoperative gastrointestinal bleeding provides new perspectives for clinicians in assessing the risk of postoperative bleeding. HRR testing of patients after PCI would be helpful in predicting the risk of gastrointestinal bleeding in advance and provide a more accurate diagnosis and treatment plan for patients. This suggestion is good news for patients, doctors, and the entire medical system. With further research and application of HRR, we expect that the incidence of gastrointestinal bleeding after PCI will be greatly reduced.

## Figures and Tables

**Table 1:** Sociodemographic characteristics.

Categories	Overall (*N* = 1647)	Q1 (*N* = 412)	Q2 (*N* = 412)	Q3 (*N* = 412)	Q4 (*N* = 411)	*p* value
Age, years, mean (SD)	69.12 (20.61)	73.01 (10.20)	68.30 (10.13)	63.82 (10.58)	57.53 (11.01)	< 0.001
Male, *n* (%)	1218 (74.0)	224 (54.4)	280 (68.0)	338 (82.0)	376 (91.5)	< 0.001
HR, bmp, mean (SD)	82.90 (9.93)	84.41 (18.97)	80.12 (16.12)	82.10 (16.38)	83.61 (18.46)	0.002
BMI, kg/m^2^, mean (SD)	30.23 (90.70)	27.50 (4.76)	34.25 (172.79)	29.40 (5.82)	29.52 (6.43)	0.334
SpO_2_%, mean (SD)	98.00 (1.41)	98.08 (1.44)	97.97 (1.40)	97.94 (1.27)	98.00 (1.52)	0.125
SBP, mmHg, mean (SD)	139.82 (24.80)	137.9 (23.76)	139.1 (25.11)	140.8 (23.67)	140.8 (24.86)	0.268
DBP, mmHg, mean (SD)	83.2 (6.50)	75.0 (14.02)	79.8 (14.28)	82.3 (13.55)	85.6 (15.36)	< 0.001
Comorbidities						
CHF, *n* (%)	504 (30.6)	182 (44.2)	130 (31.6)	112 (27.2)	80 (19.5)	< 0.001
AMI, *n* (%)	942 (57.2)	232 (56.3)	221 (53.6)	227 (55.1)	262 (63.7)	0.017
Hypertension, *n* (%)	1124 (68.2)	294 (71.4)	286 (69.4)	280 (68.0)	264 (64.2)	< 0.001
AKIP, *n* (%)	190 (11.5)	79 (19.2)	34 (8.3)	41 (10.0)	36 (8.8)	< 0.0001
Dyslipidemia, *n* (%)	879 (53.4)	161 (39.1)	213 (51.7)	239 (58.0)	266 (64.7)	< 0.0001
DM, *n* (%)	743 (45.1)	199 (48.3)	166 (40.3)	175 (42.5)	203 (49.4)	< 0.001
AF, *n* (%)	349 (21.2)	108 (26.2)	79 (19.2)	75 (18.2)	87 (21.2)	0.024
Anemia, *n* (%)	447 (27.1)	209 (50.7)	88 (21.4)	69 (16.7)	81 (19.7)	< 0.0001

*Note:* SpO_2_, pulse blood oxygen saturation; HRR: Q1 (0.98–2.86), Q2 (2.07–3.24), Q3 (3.25–3.58), and Q4 (3.59–6.91).

Abbreviations: AF, atrial fibrillation; AKI, acute kidney injury; AMI, acute myocardial infarction; BMI, body mass index; CHF, congestive heart failure; DBP, diastolic blood pressure; DM, diabetes mellitus; HR, heart rate; SBP, systolic blood pressure.

**Table 2:** Peripheral blood parameters.

Categories	Overall (*N* = 1647)	Q1 (*N* = 412)	Q2 (*N* = 412)	Q3 (*N* = 412)	Q4 (*N* = 411)	*p* value
RBC, m/uL, mean (SD)	4.5 (0.75)	3.8 (0.53)	4.5 (0.42)	4.8 (0.43)	5.2 (0.51)	< 0.0001
RDW, %, mean (SD)	13.3 (1.89)	13.9 (1.79)	13.2 (0.94)	13.1 (1.84)	12.6 (1.22)	< 0.0001
WBC, K/uL, mean (SD)	9.2 (4.58)	9.1 (4.59)	9.0 (4.21)	9.3 (4.01)	9.9 (4.25)	0.027
Platelet, K/uL, mean (SD)	224.34 (75.50)	222.9 (71.38)	223.5 (62.34)	230.8 (58.94)	238.3 (68.32)	0.0019
Hemoglobin, g/dL, mean (SD)	11.71 (2.22)	11.37 (57.12)	13.23 (10.26)	14.19 (9.68)	15.38 (11.78)	< 0.0001
TC, mmol/L, mean (SD)	2.0 (3.51)	1.6 (1.21)	2.1 (3.55)	2.0 (1.72)	2.6 (2.67)	< 0.0001
LDL, mmol/L, mean (SD)	3.2 (2.01)	2.8 (0.90)	3.1 (0.99)	3.2 (0.98)	3.4 (0.98)	< 0.0001
TG, mmol/L, mean (SD)	5.0 (3.09)	4.6 (1.24)	5.0 (1.43)	5.0 (1.35)	5.4 (1.37)	< 0.0001
HDL, mmol/L, mean (SD)	1.1 (0.30)	1.1 (0.31)	1.1 (0.25)	1.1 (0.23)	1.1 (0.25)	0.0535
Glucose, mmol/L, mean (SD)	10.3 (66.01)	27.2 (36.45)	8.5 (4.20)	8.5 (4.62)	9.5 (5.14)	0.398
BUN, mmol/L, mean (SD)	6.9 (11.21)	9.1 (6.54)	7.0 (12.93)	6.0 (1.94)	5.8 (1.90)	< 0.0001
Scr, umol/L, mean (SD)	103 (180.32)	163.3 (223.23)	82.8 (29.13)	83.2 (26.82)	81.1 (22.75)	< 0.0001
Uric acid, mmol/L, mean (SD)	389.6 (112.80)	405.5 (129.03)	381.1 (109.60)	386.2 (105.94)	398.8 (112.18)	0.0082

*Note:* RDW, red cell distribution width; TG, triglyceride; Scr, serum creatinine; HRR: Q1 (0.98–2.86), Q2 (2.07–3.24), Q3 (3.25–3.58), and Q4 (3.59–6.91).

Abbreviations: BUN, blood urea nitrogen; HDL, high-density lipoprotein; LDL, low-density lipoprotein; RBC, red blood cell; TC, total cholesterol; WBC, white blood cell.

**Table 3:** Overview of gastrointestinal bleeding within 1 year of discharge.

Categories	Overall (*N* = 1647)	Q1 (*N* = 412)	Q2 (*N* = 412)	Q3 (*N* = 412)	Q4 (*N* = 411)	*p* value
GIB, *n* (%)	20 (1.2)	8 (1.9)	6 (1.5)	4 (0.9)	2 (0.4)	< 0.0001

*Note:* HRR: Q1 (0.98–2.86), Q2 (2.07–3.24), Q3 (3.25–3.58), and Q4 (3.59–6.91).

Abbreviation: GIB, gastrointestinal bleeding.

**Table 4:** Correlation analysis of Hb, RDW, HRR, and gastrointestinal bleeding after PCI.

		Hb	RDW	HRR
GIB	Kendall's tau-b	−0.225	0.245	−0.294
*p* value		< 0.001	< 0.001	< 0.001

*Note:* The association between Hb, RDW, and HRR and gastrointestinal bleeding was analyzed by Kendall tau-b grading after PCI. The results showed that Hb, RDW, and HRR were associated with gastrointestinal bleeding.

**Table 5:** Ordered logistic regression analysis of blood parameters and gastrointestinal bleeding.

	Model 1			Model 2		
	*β*	*p*	95% CI	*β*	*p*	95% CI
Lower Hb	0.901	< 0.001	0.415∼1.400	0.801	0.042	−0.024∼1.476
Lower RDW	−1.101	< 0.001	−1.642∼−0.559	−0.725	0.031	−1.380∼−0.056
Lower HRR	1.229	< 0.001	0.719∼1.749	1.1219	0.004	0.368∼1.890

*Note:* Model 1, unadjusted; Model 2, adjusted for age, DBP, AMI; CHF, AF, DM, hypertension, dyslipidemia, anemia, AKI, WBC, RBC, platelet, TC, TG, LDL-C, serum creatinine, BUN, and uric acid.

## Data Availability

The data that support the findings of this study are available on request from the corresponding author upon reasonable request.

## Nomenclature

RDW: Red cell distribution width
GIB: Gastrointestinal bleeding
PCI: Percutaneous coronary intervention
HRR: Hemoglobin-to-red-cell distribution width ratio
CHD: Coronary heart disease
